# Mechanism and research progress of MAPK signaling pathway in myocardial fibrosis

**DOI:** 10.3389/fcvm.2025.1667568

**Published:** 2025-11-21

**Authors:** Jinqiao Wu, Min Chen, Ze Peng, Yao Sun, Juan Jin

**Affiliations:** 1 First Clinical Medical College, Heilongjiang University of Chinese Medicine, Harbin, Heilongjiang, China; 2 Department of Cardiovascular Diseases 1, The First Affiliated Hospital of Heilongjiang University of Chinese Medicine, Harbin, Heilongjiang, China

**Keywords:** MAPK, myocardial fibrosis, research progress, mechanism, drugs

## Abstract

Myocardial fibrosis (MF) is a hallmark pathological outcome of many cardiovascular diseases and a key component of cardiac remodeling. The p38 MAPK, JNK, and ERK signaling pathways are central to this process. This review summarizes the roles and interactions of these factors in MF and identifies relevant drug and non-pharmacological therapies targeting these pathways.

## 1 Introduction

Myocardial fibrosis (MF), a significant pathological basis for heart failure (HF), is characterized by excessive collagen deposition in the myocardial interstitium driven by abnormal fibroblast activation (1). These pathological changes impair systolic and diastolic functions. Three pathological subtypes are recognized: replacement fibrosis, interstitial fibrosis, and perivascular fibrosis (2). Early MF may be clinically silent and detectable only through imaging. With progression, it can lead to HF, arrhythmias, and diastolic dysfunction. Early intervention may slow disease progression; however, established MF is usually irreversible. The extent of fibrosis strongly correlates with cardiovascular mortality and hospitalization for HF. Therefore, preventive and therapeutic strategies are essential.

The mitogen-activated protein kinase (MAPK) signaling pathway is a central intracellular signal transduction network that regulates cell proliferation, differentiation, apoptosis, and stress responses (3). The MAPK pathway uses a three-kinase cascade to transmit signals. In MF, diverse pathological stimuli activate this cascade, modulate transcription factors, and alter the expression of connected genes. The principal kinases in this pathway are p38 MAPK, c-Jun N-terminal kinase (JNK), and extracellular signal-regulated kinase (ERK) (4). Furthermore, atrial fibrosis is the core pathological feature of atrial cardiomyopathy (5). Studies have demonstrated that p38 protein expression in the atrial muscle is associated with an increase in the number of myofibroblasts (6). This review synthesizes the core roles and recent progress of MAPK signaling in MF and discusses the related findings with broad relevance for understanding atrial fibrosis.

The TGF-β/Smad3 signaling pathway is also important in MF (7). TGF-β ligand binding at the cell surface triggers the phosphorylation of TGF-β receptor II, which then activates receptor I. Receptor I phosphorylates Smad2/3. Phosphorylated Smad2/3 and Smad4 can form a complex and translocates to the nucleus, affecting the expression of various profibrotic genes, including collagens (COL1A1, COL3A1, COL5A2, COL6A1, COL6A3, COL7A1) (8), PAI-1 (9), proteoglycans (10), integrins (11), connective tissue growth factor (CTGF) (12) and matrix metallopeptidase (MMP) (13). This is the classical Smad pathway. TGF-β also signals through non-Smad routes by activating MAPKs, namely p38 MAPK, JNK, and ERK, which promote MF formation.

## 2 Mechanisms of MAPK in MF, therapeutic strategies, and research progress

### 2.1 Roles of MAPK subtypes in MF

#### 2.1.1 Mechanisms of p38 MAPK promoting MF

p38 MAPK is the most extensively studied subtype. The p38 MAPK family consists of four subtypes (α, β, γ, and δ), among which p38α is the most abundantly expressed in the heart and has the closest relationship with MF. In MF, p38 MAPK is mainly activated by cytokines, growth factors, and oxidative stress. Subsequently, it promotes the development of MF by regulating processes, including inflammation, oxidative stress, apoptosis, and fibroblast activation. Lastly, it phosphorylates downstream transcription factors, enhances the production of various pro-fibrotic factors, and forms a positive feedback loop.

##### 2.1.1.1 Activation of myofibroblast differentiation

p38 MAPK is a major signaling effector pathway downstream of the TGF-β non-Smad pathway. p38 MAPK can also activate Smad3, leading to MF (14). α-smooth muscle actin(α-SMA) is the main marker protein of myofibroblasts. When cardiac fibroblasts (CFs) are activated by injury or pathological factors, such as TGF-β and angiotensin(Ang) Ⅱ, they transform into myofibroblasts expressing α-SMA, thereby enhancing collagen secretion and excessive extracellular matrix (ECM) deposition, leading to MF. Through two distinct mechanisms, p38 MAPK increases ACTA2 expression α-SMA, thereby driving cardiac fibroblast-to-myofibroblast differentiation (15).

##### 2.1.1.2 Mediation of inflammation

NF-*κ*B and AP-1 can serve as binding sites for transcription factors within the promoter region of inflammatory cytokine genes, both of which can be activated downstream of p38 MAPK. p38 MAPK can also regulate the transcription levels of pro-inflammatory factors secreted by CFs. It has been found that p38 MAPK can raise the mRNA expression of inflammatory factors in cultured human CFs (16–18). Fisetin can improve left atrial inflammation and fibrosis after myocardial infarction (MI) via the p38 MAPK signaling pathway (19).

##### 2.1.1.3 Regulation of oxidative stress

In the heart, reactive oxygen species (ROS) can activate the p38 MAPK pathway (20). Activated p38 MAPK can induce the expression of α-SMA (21). Hypoxia can also stimulate mitochondria to produce ROS, which, in turn, activate p38 MAPK. Furthermore, p38 MAPK can inhibit the function and expression of superoxide dismutase (SOD). In chronic intermittent hypoxia, p38 MAPK inhibitors can elevate SOD activity and reduce oxidative damage indicators (22). Mendelian randomization analysis further supports a negative correlation between MAPK14 (the gene encoding p38 MAPK) and SOD levels, indicating a bidirectional inhibitory relationship (23).

##### 2.1.1.4 Induction of apoptosis and myocardial remodeling

p38 MAPK can activate the expression of pro-apoptotic proteins, such as Bax and caspase-3, whereas the inhibition of p38 MAPK may result in elevated levels of anti-apoptotic proteins, such as Bcl (24, 25). These findings indicate that p38 MAPK induces cardiomyocyte apoptosis. Moreover, p38 MAPK can increase the stability of the mRNAs for MMP-1, MMP-3, and MMP-9 (26, 27). It can also induce the transcription of MMP1 (28) and MMP9 (29), leading to ECM deposition and exacerbating MF.

#### 2.1.2 Mechanisms of JNK promoting MF

JNK plays an important role in MF, and the main subtypes in the heart are JNK1/2.

##### 2.1.2.1 Activation of myofibroblast differentiation

c-JUN plays an important role in the pathological processes of MF. It can directly bind to the promoter region of collagen genes, promote ECM deposition, and amplify the inflammatory response by mediating the signal transduction of inflammatory factors, indirectly promoting MF. JNK enhances the phosphorylation of c-JUN and transcriptional activity of AP-1, elevates the levels of fibrosis-related genes, and promotes the differentiation of myofibroblasts (30). JNK inhibitors can significantly reduce the expression of fibrosis-related genes (31).

##### 2.1.2.2 Mediation of inflammation and oxidative stress

JNK stimulates NF-*κ*B and AP-1, facilitates the production of inflammatory mediators, intensifies the process of inflammation within the cardiac milieu, and promotes MF. JNK phosphorylates the N-terminus of c-JUN, enhancing its transcriptional activity and regulating collagen deposition. In hypertensive HF mice, Ang Ⅱ induction increased the phosphorylation of JNK and c-JUN nuclear translocation. These effects were eliminated by 20(S)-ginsenoside Rh2 in a dose-dependent manner (32). Moreover, Rg5 can mitigate inflammation in the hearts of mice and cultured myocardial cells by blocking the JNK/AP-1 pathway activated by Ang Ⅱ (33). Theophylline can also reduce c-JUN levels and inhibit Ang Ⅱ-induced MF (34).

##### 2.1.2.3 Regulation of extracellular matrix metabolism

JNK is a driving factor in the activation of collagen synthesis in MF. JNK can stimulate CF proliferation, leading to an increase in COL Ⅰ/Ⅲ (35). Inflammatory factors [IL-22 (36) and tryptase (37)]can stimulate JNK, resulting in enhanced collagen synthesis and fibrosis. Moreover, tryptase can also stimulate the upregulation of Fn, MMP-1, and TIMP-1 through the JNK pathway, leading to excessive ECM deposition. Blocking JNK signal transduction significantly alleviates these effects (38).

##### 2.1.2.4 Interaction with other pathways

*In vitro* studies have indicated that resistin can stimulate the production of pro-fibrotic genes via JAK2/STAT3 and JNK/c-Jun pathways. *In vivo* studies have demonstrated that overexpression of resistin significantly increases the phosphorylation of the above pathways, indicating that resistin regulates fibrosis through network pathways (31). Epigallocatechin gallate can improve MF via the TGF-β1/JNK pathway (39).

#### 2.1.3 Mechanisms of ERK promoting MF

ERK1/2 participates in cardiac remodeling and can be activated by growth factors and G protein-coupled receptor ligands (40, 41). The main types of receptors for ERK1/2 on the surface of myocardial cells are RTK (42) and GPCR (43). The binding of a growth factor to the extracellular domain of an RTK promotes tail-to-tail contact and kinase activation, which propagates signals to ERK1/2 (44).

##### 2.1.3.1 Activation of the proliferation and differentiation of cardiac myofibroblasts

Studies have demonstrated that endothelin-1 leads to the activation of fibroblasts and differentiation of myofibroblasts through the ERK1/2 signaling pathway (45). TGF-β/Smad can activate the ERK1/2 pathway, upregulate CTGF/CCN2, and promote MF (46). ERK is also a key signaling effector pathway downstream of the TGF-β non-Smad pathway. Ang Ⅱ can promote the differentiation of fibroblasts into myofibroblasts via the TGF-β1/ERK1/2 pathway, manifested as fibroblast proliferation and formation of stress fibers (47).

##### 2.1.3.2 Regulation of extracellular matrix metabolism

Hyperglycemia can activate ERK1/2 in CFs, resulting in elevated COL Ⅰ/Ⅲ mRNA and protein levels. Inhibiting ERK1/2 activation significantly diminishes collagen production (48). LPS can increase the production of MMP-2 and MMP-9 via the ERK1/2 pathway in CFs (49). TIMPs can directly inhibit MMPs and form complexes with them to regulate their activation and stability. In HF, factors such as Wnt5a and VEGF-D activate ERK signaling in CFs and myofibroblasts, resulting in the elevated production of TIMP-1 and TIMP-2 (50).

Angiotensin II type 1 receptor (AT1R) is a member of the GPCR family. When excessive Ang Ⅱ accumulates in the body and is not metabolized, it links to amino acids on the fibroblast membrane, facilitating the interaction between AT1R and different proteins, thereby activating ERK1/2 signal transduction (51). Persistent stimulation by excess growth factors and Ang II activates receptor tyrosine kinases (RTKs) and the AT1R. This drives positive feedback phosphorylation of upstream kinases and downstream effectors in the ERK1/2 pathway and promotes MF (35). An *in vitro* experiment demonstrated that stimulation with TGF-β1 can induce fibroblast proliferation, α-SMA expression, and collagen synthesis, and pretreatment with PD98059 (an ERK1/2 inhibitor) can significantly inhibit these effects (52).

### 2.2 Therapeutic strategies and research progress in regulating the MAPK signaling pathway

**Figure 1:**
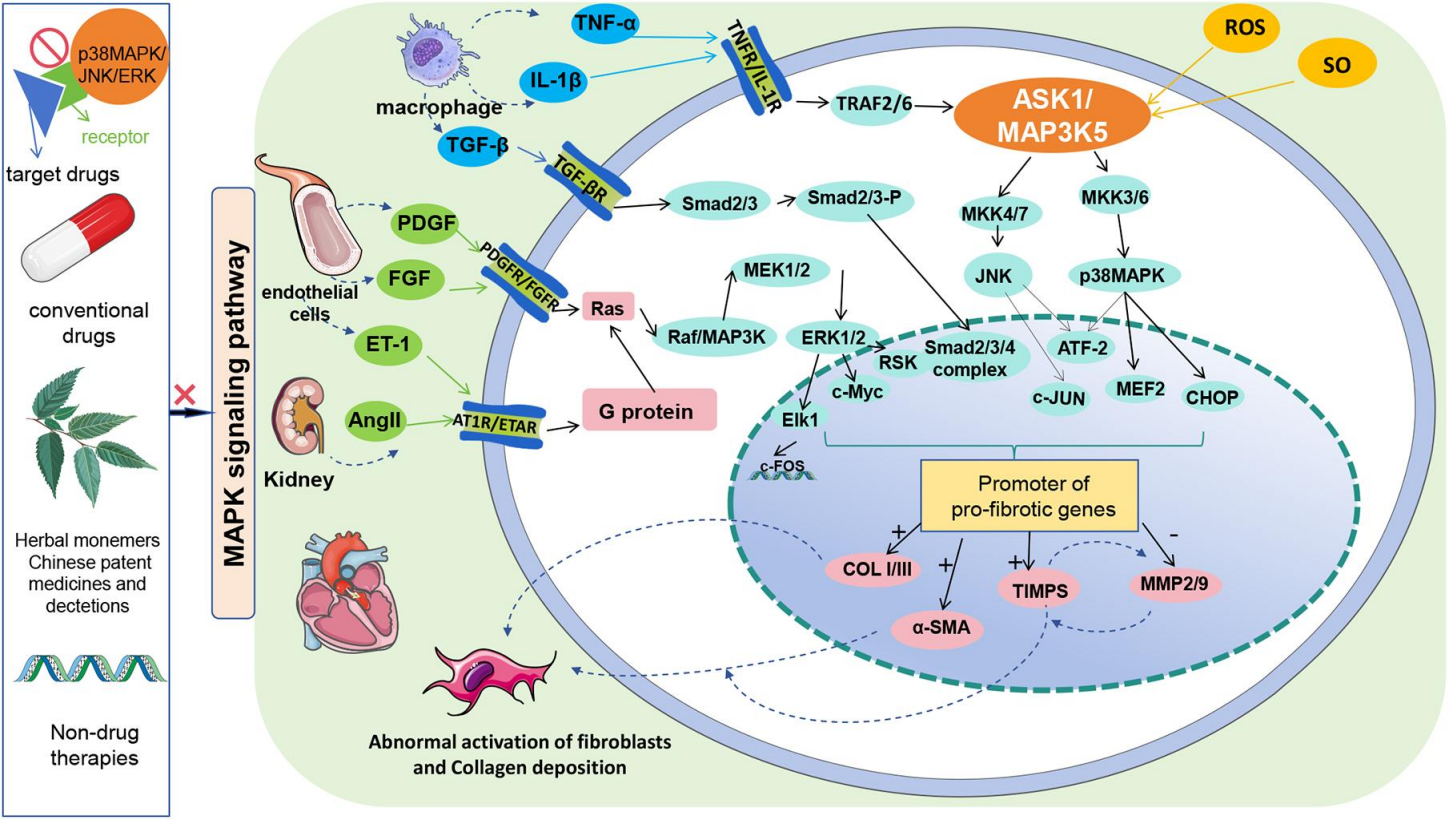
Schematic diagram of MAPK signaling pathway.

The following mechanistic studies provide a solid foundation for the treatment of MF by inhibiting the p38 MAPK/JNK/ERK signaling pathway with targeted drugs, conventional drugs, Chinese medicine monomers, Chinese patent medicines and decoctions, and non-pharmacological therapies (Figure 1). These studies provide proof-of-concept evidence for this hypothesis (Table 1).

**Table 1:** Drugs or methods to improve myocardial fibrosis by inhibiting the MAPK signaling pathway.

Name	*in vitro*, *in vivo* models	Mechanisms	pathway	References
20(S)-Rh2	Ang II induced HHF (hypertensive heart failure) mouse model	↓: p-JNK, JNK, TGF-β1, β-MyHC, COL I	JNK/AP-1	(32)
Rg5	Ang II induced HHF (hypertensive heart failure) mouse model and H9C2 cells	↓: p-JNK, β-MyHC, TGF-β1, COL I	JNK/AP-1	(33)
EGCG	STZ induced diabetes rat model	↓: TGF-β1, JNK, p-JNK, TIMP-1	TGF-β1/JNK	(39)
EVs-PAP@SA	MI mice	↓: p38α, p-p38α, TGF-β1	TGF-β1/Smad&TRPC6/NFAT	(53)
Oxymatrine	TGFβ1-induced rat CFs	↓: p-p38MAPK, p-ERK1/2, COL Ⅰ, COL III, α-SMA	p38MAPK and ERK1/2	(55)
		↑: Rate of inhibition of cell proliferation		
chidamide	Ang II induced mouse HFpEF model	↓: p38MAPK, ERK, JNK, a-SMA, Fn1, COL I, COL III, TGF-β1	MAPK	(56)
Enalapril	Ang II-induced rat CFs	↓: p-p38MAPK, ROS, TGF-β1	ROS/P38MAPK/TGF-β1	(57)
DHA	Radiation-induced myocardial fibrosis	↓: p38, α-SMA	p38/ET-1	(58)
Propranolol	Catecholamines-induced rat model of HF	↓: p38-MAPK, α-SMA, MMP-9	p38-MAPK	(59)
ivabradine	ISO-induced mouce HF model	↓: p38MAPK, α-SMA	P38-MAPK	(60)
Morroniside	Ang II-induced HCF	↓: p-JNK/p-P38, COL I, COL III, CTGF, Fn, α-SMA, MMP-2, MMP-9	p38/JNK	(61)
GLC	Diabetes fat rat model	↓: TGF-β, p-p38MAPK	TGF-β/p38MAPK	(62)
Tinglu Yixin Granules	TGFβ1—induced CFs	↓: COL I, α-SMA, p-p38MAPK, TGF-β1	TGF-β1/Smad3/p38 MAPK	(63)
zhilong Huoxue Tongyu capsule	DCM mouse model	↓: p-P38MAPK, TNF-α, α-SMA, COL I	P38MAPK	(64)
Tonifying Kidney and Activating Blood(KTBA) decoction	Rat model of HF induced by LAD ligation	↓: p-p38MAPK/p38MAPK, COL I, COL III, α-SMA	p38MAPK/NF-*κ*B/AQP4	(65)
Anshen Shumai Decoction	Rat model of HF induced by LAD ligation	↓: p-p38, EGR1, c-Fos, c-Jun, MMP-9, TGF-β2, COL Ⅰ	p38/EGR1/c-FOS/c-Jun	(66)
Guanxinning Injection	TAC induced mouse HF model	↓: p38, c-FOS, MMP1	p38/FOS/MMP1	(67)
OTUD7B	TGFβ1—induced CFs	↓: COL Ⅰ, α-SMA	FAK-ERK/P38	(68)
SMOC2	TGFβ—induced CFs	↓: p-p38, COL I, COL III, α-SMA	ILK/p38	(69)
PCI34051	ISO-induced mice	↓: p-P38, COL I, *Fn*, Ctgf mRNA, α-SMA, TGF-β1 mRNA	p38 MAPK	(70)
Nobiletin	Rat AMI model established by LAD ligation	↓: JNK, P-JNK	JNK	(73)
C66	LAD ligation induced mouse MI model	↓: TGF-β1, α-SMA	JNK	(74)
Dan-shen Yin	MI model established by LAD ligation	↓: p-JNK, p-ERK, TGF-β, COL I, COL III, α-SMA, MMP2, MMP9	PI3K/AKT, MAPK, TGFβ/Smad3	(75)
ONSMP	Rat HF model with LAD ligation	↓: p-ERK1/2, pJNK1/2, COL I/III, α-SMA	JNK/ERK	(76)
Knock down SK2	Ang II-induced CFs	↓: p-Smad2/3, p-p38, p-ERK1/2, p-JNK, TGF-β1, COL I, α-SMA	Smad2/3, MAPK	(77)
TMEM100	TAV mouse	↓: Col1a1, Col3a1, Col8a1, Ctgf	TAK1-JNK/p38	(78)
Silence HOTAIR	Ang II treated atrial fibroblasts	↓: α-SMA, COL I/III, CTGF, p-ERK/p-JNK	Wnt5a, ERK/JNK	(79)
PAK1	Ang II-induced CFs	↓: α-SMA, COL I, p-JNK, p-c-Jun	JNK/c-Jun	(80)
GPR30 Agonist G1 Co-Administration	TAC induced elderly female mice	↓: p-ERK, ERK, MMP-9, TGF-β1	ERK	(82)
Lenalidomide	ISO-induced MF	↓: ERK, Fn, COL I, COL III, α-SMA	ERK	(83)
Miglustat	ISO-induced CFs	↓: p-ERK, CTGF, PCNA, Fn, COL I, COL III, α-SMA	ERK, STAT3, Akt, GSK3β	(84)
Vericiguat	Ang II-induced MF	↓: ERK1/2, p38-MAPK, Fn, Col1α1, Col3α1, TGF-β1	ERK1/2, p38-MAPK	(85)
GPS-1-1	TGF-β1induced CFs	↓: p-JNK, p-ERK, α-SMA, COL I, COL III, Fn, Vim	MAPK/PI3K/AKT	(87)
paeonol	TAC induced mouse HF model	↓: COL I, CTGF, Fn-1	ERK1/2/JNK	(88)
Qifu yixin prescription	TAC mouse and Ang II-induced CFs	↓: p-ERK, COL I, α-SMA	sGC/PKG/ERK	(89)
ONSMP	Rat HF model with LAD ligation	↓: p-ERK1/2, c-Fos, COL I/III, α-SMA	RAS/RAF/MEK/ERK	(90)
miR-450a-2-3p agomir	Mouse model treated with ISO and miR-450a-2-3p agomir	↓: ERK1/2, α-SMA, COL I, COL3	miR-450a-2-3p/ERK(1/2)	(91)
SO_2_	Ang II-induced mouse	↓: ERK ½	ERK1/2	(92)

#### 2.2.1 Agents and approaches acting on the p38 MAPK pathway

##### 2.2.1.1 P38 MAPK-targeted drugs

SB203580 is a selective p38 MAPK inhibitor. Studies have found that SB203580 can reduce the expression of α-SMA, COL Ⅰ, TGF-β1, and TNF-α, and inhibit the progression of MF. Novel delivery systems, such as hydrogels loaded with p38α antagonistic peptides or nanoparticles targeting activated fibroblasts, enhance the local efficacy of anti-fibrotic drugs (53, 54). Oxymatrine simultaneously inhibits the TGF-β1 and p38 MAPK pathways. Compared with SB203580 alone, this combination produced a significantly stronger anti-fibrotic effect, indicating synergistic inhibition (55).

##### 2.2.1.2 Conventional drugs targeting the p38 MAPK

In the Ang Ⅱ-induced HFpEF mouse model, chidamide improved myocardial hypertrophy and fibroblast proliferation and differentiation by inhibiting p38 MAPK. Subsequent studies have demonstrated that chidamide administration markedly diminishes the expression of cardiac fibrosis markers (PCNA, COL Ⅰ, COL Ⅲ, TGF-β1, and α-SMA) (56). Enalapril can inhibit the proliferation of Ang Ⅱ-induced rat fibroblasts through the ROS/p38 MAPK/TGF-β1 pathway (57). Liu found that DHA can reduce collagen deposition and α-SMA expression via blocking the p38/ET-1 pathway in cardiomyocytes, and alleviate radiation-induced MF (58). Propranolol can alleviate MF caused by excessive catecholamines by modulating p38 MAPK (59). Sun found that ivabradine exhibits a protective effect against isoproterenol-induced cardiac injury, which is related to its blocking of the p38 MAPK signaling pathway, reduction of MF, decrease in cardiomyocyte apoptosis, and increase in autophagy (60).

##### 2.2.1.3 Chinese medicine monomers targeting the p38 MAPK

Oxymatrine can inhibit TGF-β1-induced cardiac fibroblast proliferation and fibroblast-myofibroblast transformation by modulating p38 MAPK and ERK1/2 pathways (55). Zheng found that Morroniside, the active ingredient of Fructus Corni, can block the p38/JNK pathway by downregulating KLF5, which can improve the proliferation, migration, and extracellular matrix deposition of CFs, thereby exerting a protective effect on MF (61). GLC can ameliorate MF in murine models of diabetic cardiomyopathy via the TGF-β/p38 MAPK pathway (62).

##### 2.2.1.4 Chinese patent medicines and decoctions targeting the p38 MAPK

Tinglu Yixin Granules can inhibit the TGF-β1/Smad3/p38 MAPK signal transduction, reduce the transformation of fibroblasts, and inhibit the expression of collagen and α-SMA. *In vivo* experiments have also confirmed that Tinglu Yixin Granules can ameliorate MF in diabetic mouse models (63). Zhilong Huoxue Tongyu Capsules can improve MF animals suffering from diabetic cardiomyopathy. The results of enrichment analysis point to MAPK. *In vivo* experiments have confirmed that Zhilong Huoxue Tongyu Capsules downregulate the expression of fibrosis-related proteins (64). In a study of the HF rat model with left anterior descending ligation, Xu found that Tonifying Kidney and Activating Blood Decoction can regulate the p38 MAPK/NF-*κ*B/AQP4 axis to delay MF (65). Another study found that Anshen Shumai Decoction mainly exerts its effect by down-regulating the gene expression of FOS and EGR1 and the p38 MAPK pathway, which significantly inhibits myocardial cell apoptosis and MF in infarcted rats (66). Furthermore, Guanxinning Injection inhibits myocardial hypertrophy and fibrosis in HF mice by modulating the p38/FOS/MMP1 pathway (67).

##### 2.2.1.5 Non-pharmacological therapies targeting the p38 MAPK

OTUD7B is a drug target that can mitigate MF by decreasing the phosphorylation of ERK/p38. Research indicates that silencing OTUD7B with siRNA increases the levels of α-SMA and COL Ⅰ in CFs, while overexpressing OTUD7B with adenovirus reduces their expression, thereby producing an anti-fibrotic action (68). Therapeutic SMOC2 silencing inhibits the production of COL Ⅰ, COL Ⅲ, and α-SMA through the ILK/p38 pathway *in vitro (*69). Moreover, the delivery of mBMSCs-EVs and PAP using SA hydrogel mitigated MF in mice with MI (53). Treatment with PCI34051 suppresses the expression of murine fibrosis markers by diminishing isoproterenol-induced activation of p38 MAPK (70).

#### 2.2.2 Agents and approaches acting on the JNK pathway

##### 2.2.2.1 JNK's targeted drugs and conventional drugs

Studies have demonstrated that SP600125 (a JNK inhibitor) can alleviate MF by inhibiting JNK phosphorylation, reducing apoptosis, and decreasing oxidative stress in animals and cells (71). In a murine model of MI, SP600125 can modulate the JNK pathway and counteract the pro-fibrotic effect of mCRP (72). MicroRNAs targeting the JNK pathway can alleviate hypoxia-induced MF by reducing collagen expression, further supporting the therapeutic potential of the JNK pathway (38). Guì et al. found that epigallocatechin gallate can improve MF in streptozotocin-diabetic rats with intraperitoneal injection by modulating the TGF-β1/JNK signaling pathway (39).

##### 2.2.2.2 Chinese herbal monomers targeting the JNK

Nobiletin improves MF in a rat model of acute myocardial infarction (AMI) by inhibiting the JNK pathway. It was also found that intraperitoneal injection of nobiletin at a medium dose exhibited the best improvement effect (73). C66, a curcumin analog, can improve MF in rats following AMI by inhibiting the JNK pathway (74). 20(S)-Ginsenoside Rh2 [20(S)-Rh2] is a chemical present in Radix Ginseng and studies have found that it can ameliorate MF in hypertensive rats and those with HF induced by Ang II, as well as in ventricular myocytes. Studies have demonstrated that 20(S)-Rh2 can inhibit the levels of TGF-β1, β-MyHC, and COL Ⅰ in model rats and ventricular myocytes in a dose-dependent manner at both the transcriptome and proteomic levels by inhibiting JNK/AP-1 (32). Furthermore, Ginsenoside Rg5 can mitigate Ang Ⅱ-induced MF by suppressing the JNK/AP-1 pathway (33).

##### 2.2.2.3 Chinese patent medicines and decoctions targeting the JNK

Danshen Yin can diminish the expression of COL Ⅰ, COL Ⅲ, α-SMA, MMP 2, MMP9, and TGF-β by inhibiting JNK and ERK pathways, thereby alleviating MF following MI in rats (75). Optimized new Shengmai powder (ONSMP) ameliorated MF in rats with HF. The study found that medium and high doses of ONSMP reduced the expression levels of serum p-ERK1/2 and p-JNK1/2 in the myocardial tissue of rats with HF (76).

##### 2.2.2.4 Non-pharmacological therapies targeting the JNK

Research has found that SK2 channel knockdown can inhibit the differentiation of fibroblasts and the secretion of collagen induced by Ang Ⅱ. Moreover, knocking down SK2 significantly inhibits the activity of signaling molecules associated with the TGF-β pathway, resulting in a substantial reduction in the phosphorylation levels of Smad2/3, p38, ERK1/2, and JNK (77). Zhang's research found that the overexpression of transmembrane protein 100 can alleviate MF in mice with transverse aortic constriction via the TAK1-JNK/p38 pathway (78). HOTAIR silencing exhibits anti-migratory and anti-proliferative effects on primary atrial fibroblasts by inhibiting the Wnt5a/ERK/JNK pathway (79). Moreover, Zhou found that decreased PAK1 expression can inhibit Ang Ⅱ-induced proliferation, migration, and differentiation of HCFs through the JNK/c-Jun pathway (80).

#### 2.2.3 Agents and approaches acting on the ERK pathway

##### 2.2.3.1 ERK-targeted inhibitors

In the cardiac transplantation paradigm, ERK inhibition using U0126 attenuates graft fibrosis (81). PD98059 can reduce the levels of phosphorylated ERK1/2, MMP-9, and TGF-β1 in CFs, demonstrating its ability to resist MF (82).

##### 2.2.3.2 Conventional drugs modulating the ERK pathway

Lenalidomide inhibits β-adrenergic receptor-induced MF by diminishing the gene and protein expression of Fn, COL Ⅰ, COL Ⅲ, and α-SMA via the PI3K/AKT and JNK pathways (83). Moreover, Miglustat can improve β-adrenergic receptor-induced MF by partially inhibiting the ERK pathway (84). Studies have determined that high-dose vericiguat can significantly improve Ang Ⅱ-induced left ventricular MF in murine models. This effect is accomplished by regulating the ERK1/2 or p38 MAPK pathway to attenuate the expression of Col1a1, Col3a1, and Tgfb1 (85). Tetrandrine improves aortic constriction-induced MF in mice via the MAPK/NF-*κ*B pathway, where MAPK mainly refers to JNK and ERK pathways (86).

##### 2.2.3.3 Chinese medicine monomers targeting the ERK

*In vitro* experiments have confirmed that GSP-1-1 inhibits the activation of the MAPK/PI3K/AKT signaling pathway by downregulating the expression of PDGFB and reducing the protein expression of Vim, Fn, α-SMA, COL Ⅰ, and COL Ⅲ, thereby inhibiting the fibrosis of fibroblasts (87). *In vivo* experiments have indicated that paeonol can inhibit fibrosis in TAC-induced HF mice via the ERK1/2/JNK pathway (88).

##### 2.2.3.4 Chinese patent medicines and decoctions modulating the ERK pathway

The Qifu Yixin prescription worsens MF by activating sGC to suppress ERK phosphorylation, thereby slowing the progression of HF in mice caused by transverse aortic constriction (TAC) (89). ONSMP can diminish MF in HF mice (90).

##### 2.2.3.5 Non-pharmacological interventions targeting the ERK pathway

miR-450a-2-3p overexpression can reduce the elevation of ISO-induced α-SMA, COL Ⅰ and COL Ⅲ by lowering ERK1/2, indicating that it can inhibit collagen formation in cardiac tissue via the ERK pathway (91). Sulfur dioxide can inhibit cardiac fibroblast proliferation by sulfenylating ERK1/2 and phosphorylating ERK1/2 (92). Wang et al. found that overexpression of GPR30 and combined administration of its agonist G1 can reduce MF induced by TAC in aged female mice (82).

## 3 Conclusion and future perspectives

The MAPK-p38 MAPK/JNK/ERK pathway is significant in MF. This process is triggered by stimuli, such as growth factors, initiating MF in a cascading manner. This process involves mechanisms such as oxidative stress, signal transduction, gene expression, and extracellular matrix deposition. Fibroblasts can proliferate and differentiate into myofibroblasts via the MAPK pathway, and the intervention of drugs on this pathway is significant. These findings indicate that MAPK serves as a key target driving MF. Our study implicates p38 MAPK in atrial fibrosis, suggesting that MAPK signaling may be a shared mechanism linking myocardial and atrial fibrosis. As atrial fibrosis sustains atrial fibrillation (AF), targeting this pathway could delay or reverse fibrosis and offer a disease-modifying approach to AF. If MAPK proves to be a shared pathway, the targeted drugs developed could also delay or reverse atrial fibrosis, offering a potential avenue for fundamental treatment of AF.

This review summarizes the therapeutic modalities that regulate MAPK signaling. These include targeted agents, conventional drugs, Chinese patent medicines, and non-pharmacological interventions. Across studies, these approaches reduce myocardial hypertrophy, limit fibroblast proliferation and differentiation, decrease collagen deposition, and suppress MF. In our study, morroniside and oxymatrine acted on multiple pathways and provided synergistic benefits against MF. Compound herbal formulations can also slow down MF progression by simultaneously regulating MAPK, inflammation, and apoptosis. Combination therapy is appropriate for multi-target regulation using herbal compounds. Pairing MAPK inhibitors with other anti-fibrotic agents can also allow dose reductions and may help prevent drug resistance.

Furthermore, p38 MAPK, JNK, and ERK have various subtypes, and the expression scenarios of these subtypes are also different. For example, the primary subtype of p38 MAPK expressed in the myocardium is p38α. The development of subtype-selective inhibitors should be a key goal for future studies. Disease heterogeneity limits the generalizability of treatment effects, and different etiologies activate distinct MAPK pathways. For instance, MF in diabetic cardiomyopathy is marked by elevated p38 activity, whereas JNK and ERK are more active in HF models. These differences support individualized or stratified anti-fibrotic strategies across diseases. Most current studies have emphasized oral agents. Although oral dosing is convenient, it often fails to achieve adequate drug concentrations in target tissues. Cardiac or fibroblast-targeted nanocarriers and ligand-guided delivery systems can increase early cardiac drug levels while reducing systemic exposure and toxicity.

In mice with MI, a fibronectin gel-loaded Gouqi-derived nanovessel can target the p38-MAPK signaling pathway to attenuate myocardial cell apoptosis and limit the progression of MF (93). In preclinical studies, CRISPR/Cas9 gene-editing technology has been proven to effectively correct genetic mutations in hypertrophic cardiomyopathy and dilated cardiomyopathy, reduce MF, and improve heart function, indicating the potential of this technology in treating hereditary cardiomyopathies (94). Furthermore, in *in vitro* experiments, MicroRNA29a can inhibit the proliferation of fibroblasts by targeting ERK1/2 (95), and miR-43 (14), miR-32-5p (96), miR-338-3p (97), and miR-155 (98) can affect the activation of the MAPK pathway and regulate the differentiation of fibroblasts. These findings illustrate the potential of selectively targeting CFs.

With the advancement of technology and an in-depth understanding of the mechanism, the MAPK pathway remains an attractive target for MF research. This review examines the fundamental role of the MAPK pathway in MF and introduces relevant drugs. There is a solid scientific basis for targeting the MAPK pathway in MF.

Although preclinical studies of MAPK-targeted therapy for MF are encouraging, the current evidence has clear limitations. Most reports are short-term animal or cellular experiments. These designs cannot predict drug effects across the entire disease course, and *in vitro* systems do not replicate the physiological complexity of humans. Research also focuses on a few key nodes within MAPK; accordingly, its role in the broader disease network remains incomplete. To close these gaps, future studies should prioritize well-designed phase I/II clinical trials, include long-term follow-up endpoints, and comprehensively assess durability and long-term risks. These findings would provide clinicians with more precise and diverse treatment options.
